# On the combination of adaptive neuro-fuzzy inference system and deep residual network for improving detection rates on intrusion detection

**DOI:** 10.1371/journal.pone.0278819

**Published:** 2022-12-12

**Authors:** Jia Liu, Wang Yinchai, Teh Chee Siong, Xinjin Li, Liping Zhao, Fengrui Wei

**Affiliations:** 1 Faculty of Computer Science and Technology, University Malaysia Sarawak, Sarawak, Malaysia; 2 Faculty of Big Data, Weifang Institute of Technology, Weifang, China; 3 Faculty of Cognitive Sciences and Human Development, University Malaysia Sarawak, Sarawak, Malaysia; 4 College of Control Science and Engineering, China University of Petroleum, Qingdao, China; University of Wisconsin-Eau Claire, UNITED STATES

## Abstract

Deep Residual Networks (ResNets) are prone to overfitting in problems with uncertainty, such as intrusion detection problems. To alleviate this problem, we proposed a method that combines the Adaptive Neuro-fuzzy Inference System (ANFIS) and the ResNet algorithm. This method can make use of the advantages of both the ANFIS and ResNet, and alleviate the overfitting problem of ResNet. Compared with the original ResNet algorithm, the proposed method provides overlapped intervals of continuous attributes and fuzzy rules to ResNet, improving the fuzziness of ResNet. To evaluate the performance of the proposed method, the proposed method is realized and evaluated on the benchmark NSL-KDD dataset. Also, the performance of the proposed method is compared with the original ResNet algorithm and other deep learning-based and ANFIS-based methods. The experimental results demonstrate that the proposed method is better than that of the original ResNet and other existing methods on various metrics, reaching a 98.88% detection rate and 1.11% false alarm rate on the KDDTrain+ dataset.

## Introduction

Nowadays, leading technologies increase cyber risks for users and businesses. And, according to the *Cisco Annual Internet Report (2018–2023) White Paper* [1], the threat of network intrusions is growing year by year. There was a 776% growth in attacks between 100 Gbps and 400 Gbps from 2018 to 2019. Over half of the operators experienced infrastructure outages. The advance in technologies, such as e-commerce, mobile payments, cloud computing, Big Data and analytics, IoT, AI, machine learning, and social media, is the main driver of economic growth but has also led to a higher incidence of cyberattacks [1]. As one of the key technologies for ensuring network security, intrusion detection plays a more and more important role. However, under the changing network environment, new intrusion detection technologies need to be studied.

Deep learning algorithms bring new probabilities for intrusion detection. Deep learning algorithms can capture highly complex underlying patterns in the data. And, due to the great performance of deep learning in image recognition, various deep learning algorithms have been proposed. And, many of them have been applied in intrusion detection systems (IDSs), like artificial neural networks (ANN) [2], Long Short-Term Memory (LSTM) [3], and Convolutional Neural network (CNN) [4]. However, few studies have successfully applied ResNet to intrusion detection. This is not only because the structure of ResNet is complex, but also because the ResNet algorithm is prone to overfit on problems with uncertainty, such as IDS problems.

**Fig 1:**
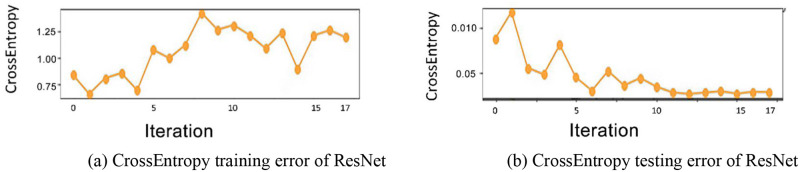
Evaluation results of ResNet on KDDTrain+ dataset. The subfigure (a) denotes the CrossEntropy training error of ResNet among epochs. The subfigure (b) denotes the CrossEntropy testing error of ResNet among epochs.

**Fig 2:**
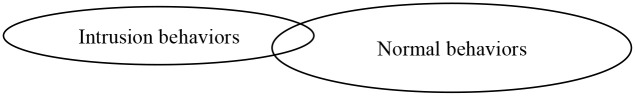
The Overlap between Intrusion Behaviors and Normal Behaviors.

We evaluate the performance of ResNet on the NSL-KDD (National security lab–knowledge discovery and data mining) dataset. Although, ResNet performs very well on image recognition, reaching a 4.92% error rate [5]. And it is considered one important breakthrough to help train deeper networks to recognize deeper patterns. However, when we trained and tested ResNet on intrusion detection’s benchmark dataset NSL-KDD dataset (including the KDDTrain+ dataset and KDDTest+ dataset, some intrusion types only exist in the KDDTest+ dataset), the evaluating results shows a serious overfitting problem of ResNet on this dataset, as shown in Fig 1. Actually, Y. Xiao and X. Xiao [6] once pointed out that ResNet is prone to overfitting for low-dimensional and small-scale datasets, resulting in reduced generalization ability of the model, in 2019. And we think this is one of the reasons, the other reason is that the ResNet is easy to overfit on problems with uncertainty due to the too-detailed classification of ResNet. However, intrusion detection problems are fuzzy classification problems as shown in Fig 2. Thus, to address this problem, we improve the ResNet with fuzzy logic in this study and propose a new architecture for intrusion detection problems. The contributions of this study can be summarized below:

- First, we propose a new and deep architecture for intrusion detection problems. This architecture enables continuous attributes to keep uncertainty property in the deep training processes.
- Second, to identify deeper patterns in the training data, we use ResNet to train the model with the fuzzy rules generated by ANFIS and concrete attributes. Meanwhile, to optimize the model, we provide a mechanism that connects the ANFIS and ResNet to co-train the two algorithms with the losses.
- Third, to evaluate the performance of the proposed method, we realize the proposed method with Python 3.7, PyTorch, and sklearn on the NSL-KDD dataset. The results of the experiment show improved performance compared with ResNet and other methods. This means the alleviation of ResNet’s overfitting problem on problems with uncertainty.

The remainder of this paper is organized as follows. In Section II, we review the related research in the field of intrusion detection, especially how ANFIS-based and Deep learning-based methods facilitate the development of intrusion detection. Section III gives a brief introduction to Pearson correlation analysis, K-means, ANFIS, and ResNet. In Section IV, a description of the proposed method is introduced. Then, Section V highlights the proposed method with a discussion of the experimental results and a comparison with a few previous studies on the NSL-KDD dataset. Finally, the conclusions are discussed in Section VI.

## Related work

Deep learning has demonstrated its effectiveness in dimensionality reduction and classifying missions. It was proposed by Hinton [7] based on the deep belief network (DBN) in 2006. And, the first real multilayer structure learning algorithm, the convolution neural network (CNN), is proposed by LeCun et al. in [8], which utilizes spatial relative relations to reduce the number of parameters and improve training performance. Then, many different architectures of deep learning such as Convolutional Neural Networks (CNNs), Recurrent Neural Networks (RNNs) have been proposed and achieved success in various fields, such as image and video recognition, audio processing, natural language processing, autonomous systems, and robotics [9].

In the field of intrusion detection, deep learning also has received special attention in recent years. The following describes recent research in intrusion detection and categorizes them according to the different basic architectures.

DBN has proved to be the most influential deep and generative neural network that learns one layer of features from unlabeled data. It has been widely studied in intrusion detection such as [10–13]. For example, Y. Zhang, Li, & Wang [14] proposed an improved Genetic Algorithm (GA) and DBN. This algorithm uses GA to optimize the number of hidden layers and the number of neurons in DBN. Therefore, the architecture of DBN is optimized and achieved a higher detection rate.

Numerous ANN-based approaches such as [2,15–19] are applied in IDSs. For instance, in [20], G. Wang, Hao, Mab, & Huang presented a fuzzy clustering enhanced ANN, FC-ANN, which uses fuzzy clustering to generate different training subsets of ANNs. Then, a fuzzy aggregation module is finally implemented to aggregate ANNs’ results. In their IDS approach, they have applied ANN, fuzzy clustering, and Fuzzy aggregation to recognize intrusions accurately. Likewise, in another ANN-based IDS scheme, Ashfaq, etc. In [21], the authors presented a fuzzy semi-supervised ANN, which is able to be trained through the combination of labeled and unlabeled samples. The divide and conquer strategy (data categorized into different groups according to the magnitude of fuzziness) is used for categorizing the unlabeled samples and incorporating each category with the original training set. The ANN is implemented to output a fuzzy membership vector and retrain the conquering category. The authors claimed that the unlabeled sample belonging to fuzziness groups makes major contributions to improving the ANN’s performance compared with the existing classifier. In [22], the authors applied ANN with a multiverse optimizer (MVO, a new natural evolutionary algorithm). They used MVO to train an ANN to identify new attacks. Furthermore, they conducted the experiment on both NSL-KDD and the new benchmark dataset UNSW-NB15. The authors demonstrated that their solution has better performance than PSO-ANN, et al.

Various architectures of RNN were implemented in IDSs because of RNN’s ability to extract the temporal features from the input data, such as [19,23–25]. For example, the IDS scheme introduced in [12], proposed a two-level LSTM model for self-adaptive detection of 5G mobile networks. First, the first level of the approach is a supervised or semi-supervised learning approach that enables DBN or Stacked AutoEncoders (SAE) to run as fast as possible on each Radio Access Network (RAN). Then, all the collected symptoms from the first layer are sent to the Network Anomaly Detection (NAD) component, where they are assembled and used as input for an LSTM Recurrent Network. The authors have applied a well-known botnet dataset CTU for training and validating. They also showed that the architecture can self-adapt the anomaly detection system based on the volume of network flows and optimize resource consumption.

The IDS schemes presented in [26] and [25], proposed CNN-based IDSs. In [26], the authors take traffic data as images to train on CNN. In [25], the authors use CNN to extract meaningful features from IDS big data. And they use weight-dropped LSTM to retain long-term dependencies among extracted features to prevent overfitting problems.

Less ResNet-based IDSs are designed to deal with intrusions. The ResNet-based scheme presented in [27], converts the network traffic data into image form and trained a ResNet over the converted data. However, this performance is only in the case of binary classification. In [6], Yuelei Xiao and Xing Xiao presented a simplified residual network (S-ResNet) and tested it on the NSL-KDD dataset. They used metrics such as accuracy and F1-score for performance analysis. This scheme’s most important attributes are its capability to prevent ResNet’s overfitting problem for low-dimensional and small-scale datasets by simplifying the network structure.

**Fig 3:**
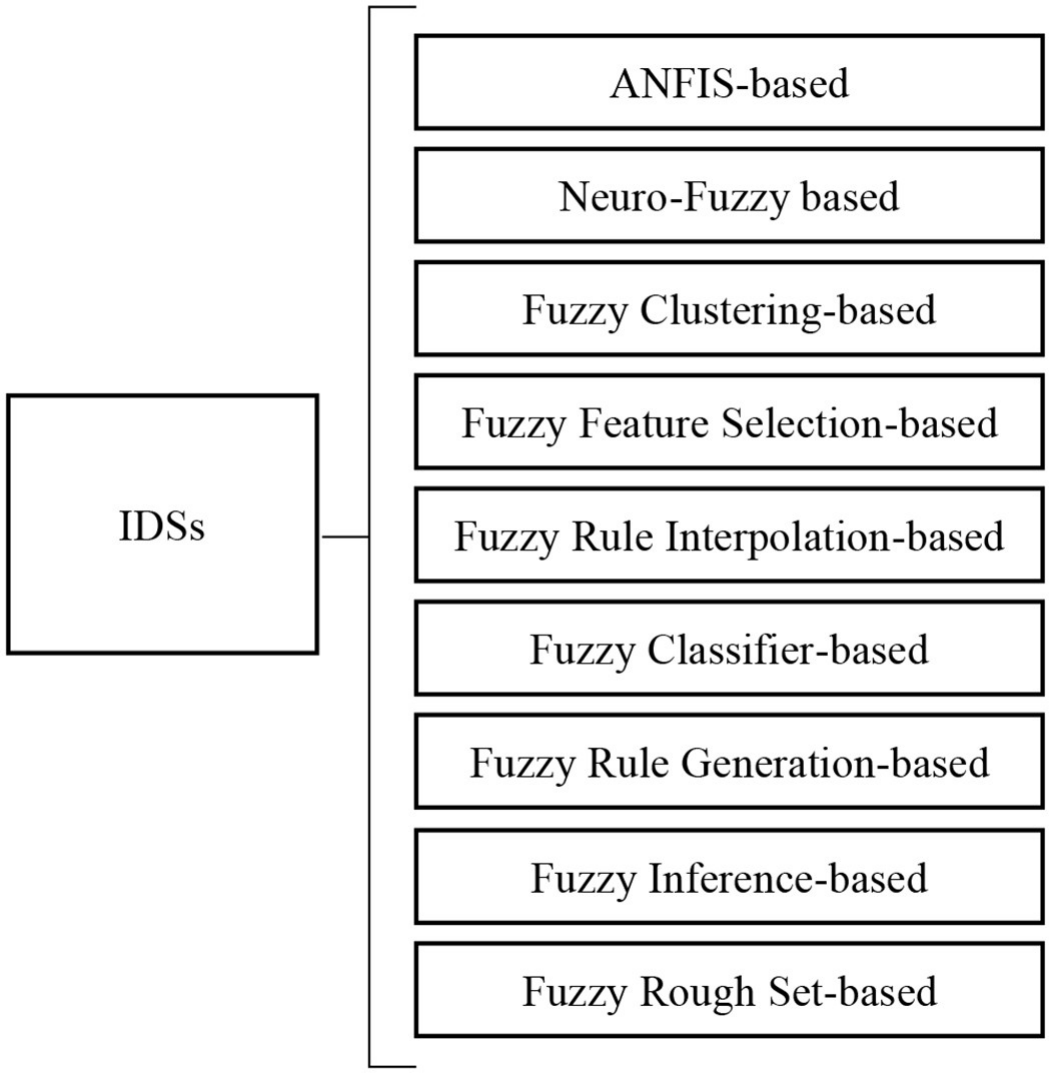
Classification of fuzzy IDSs. The 9 categories of fuzzy intrusion detection schemes according to [29].

Fuzzy logic is an effective method to provide fuzziness in attributes and reduce the overfitting problem of algorithms. Norbert Wiener, the founder of cybernetics, once pointed out that man’s superiority over the most perfect machine is that man is capable of using fuzzy concepts [28]. This shows that there is an essential difference between the human brain and the computer. Thus, if we want to simulate the human brain, fuzzy logic is essential. Fuzzy schemes have successfully proved their ability to detect intrusions and malicious behaviors in the presence of uncertain data [29]. Therefore, a large number of fuzzy approaches have been successfully applied in IDSs. And, Mohammad Masdari and Hemn Khezri [29] categorized various fuzzy intrusion detection schemes into nine categories in 2020, as shown in Fig 3. Meanwhile, they illustrated that the ANFIS classifier is one mostly used classifiers in various misuse detection schemes studies.

ANFIS algorithm is an algorithm that combines the uncertainty processing ability of fuzzy logic with the learning process of the ANNs. ANFIS was first used in IDSs in 2007. Toosi & Kahani [30] used five ANFIS modules to explore intrusive activity, reaching a 95.3% detection rate on the KDDCUP99 dataset. Then, Chan et al. [31] presented a policy-enhanced fuzzy model with ANFIS characteristics. Devi et al. [32] introduced an IDS scheme using ANFIS to detect security attacks on 5G wireless networks.

In the combination of ANFIS and other algorithms, D. Karaboga and E. Kaya [33] proposed a hybrid artificial bee colony (ABC) algorithm to train ANFIS. This algorithm uses arithmetic crossover to quickly converge r and has better efficiency than the standard ABC algorithm. Altyeb Altaher presented an IDS scheme EHNFC in [34], which is an evolutionary neuro-fuzzy classifier for malware classification. It can use fuzzy rules to detect fuzzy malware and improve its detection accuracy by learning new fuzzy rules to evolve its structure. In addition, it uses an improved fuzzy rule updating clustering method to update the centroid and radius of the clustering permission feature. These changes to the application of clustering methods improve the convergence of clustering and create rules that adapt to the input data, thereby improving the accuracy. The scheme introduced in [35] adopts data mining methods such as neural fuzzy and radial basis support vector machine to achieve a high detection rate when dealing with security attacks. The method is mainly divided into four stages, and k-means clustering is used to generate parameter-tuning subsets. Based on these subsets, various neural fuzzy models are trained to form classification vectors of support vector machines.

For solving the problem of the large volume of data resulting in the network getting expanded with false alarm rate of intrusion and detection accuracy decreased, Manimurugan, Majdi, Mohmmed, Narmatha, & Varatharajan [36] presented an algorithm CSO-ANFIS. This algorithm uses the Crow Search Optimization algorithm to optimize ANFIS and reaches a 95.8% detection rate on the NSL-KDD dataset.

ANFIS is widely used and studied in intrusion detection systems and misuse detection systems. However, ANFIS only has five layers in the original algorithm, which will reduce the deeper feature extraction ability of the algorithm. Therefore, the combination of ANFIS and deeper networks for deeper feature extraction lacks studies.

Thus, nowadays, deep learning has become more and more attractive in the intrusion detection field, and many deep learning-based models have been applied to intrusion detection. Meanwhile, in various deep learning-based models, ResNet has attracted more and more attention due to its deeper feature extraction ability. However, fewer ResNet-based methods are applied in the IDSs because of the limitations mentioned above. To alleviate the overfitting problem of ResNet on intrusion detection problems and apply ResNet effectively in intrusion detection problems, fuzzy logic is a potential solution.

## Preliminaries

### Pearson correlation analysis

The Pearson correlation coefficient *r* is usually used to measure whether there is a linear relationship between two objects, as shown below:

$${\text{r}}_{xy}=\frac{\sum \left(x-\bar{x}\right)(y-\bar{y})}{\sqrt{\Sigma_{1}^{n}{\left(x_{i}-\bar{x}\right)}^{2}}\sqrt{\Sigma_{1}^{n}{\left(y_{i}-\bar{y}\right)}^{2}}}$$ (1)

The *r* describes the degree of linear correlation between *x* and *y*. Usually, *r* > 0 indicates *x* and *y* are positively correlated. *r* < 0 indicates *x* and *y* are negatively correlated. The larger the absolute value of *r*, the stronger the correlation. In normal circumstances, the correlation strength of the attribute is judged by the following value range: 0.8–1.0 very strong correlation, 0.6–0.8 strong correlation, 0.4–0.6 moderate correlation, 0.2–0.4 weak correlation, 0.0–0.2 very weak correlation or no correlation.

### K-means algorithm

K-means is one of the most popular clustering algorithms because it is very flexible, simple, intuitive, easy to implement, and fast in execution [37]. The main steps of the K-means clustering algorithm are as follows:

**Stage 1** According to the data ranges of *n* data objects, *k* cluster centers are randomly initialized.
**Stage 2** Assign each object to the group closest to the center.
**Stage 3** The location of each center is updated by calculating the average of the objects assigned to it.
**Stage 4** Stage 2 and Stage 3 are repeated until the maximum number of iterations is reached, or until the cluster, center is no longer moving.

### Adaptive Neuro-Fuzzy Inference System-ANFIS

**Fig 4:**
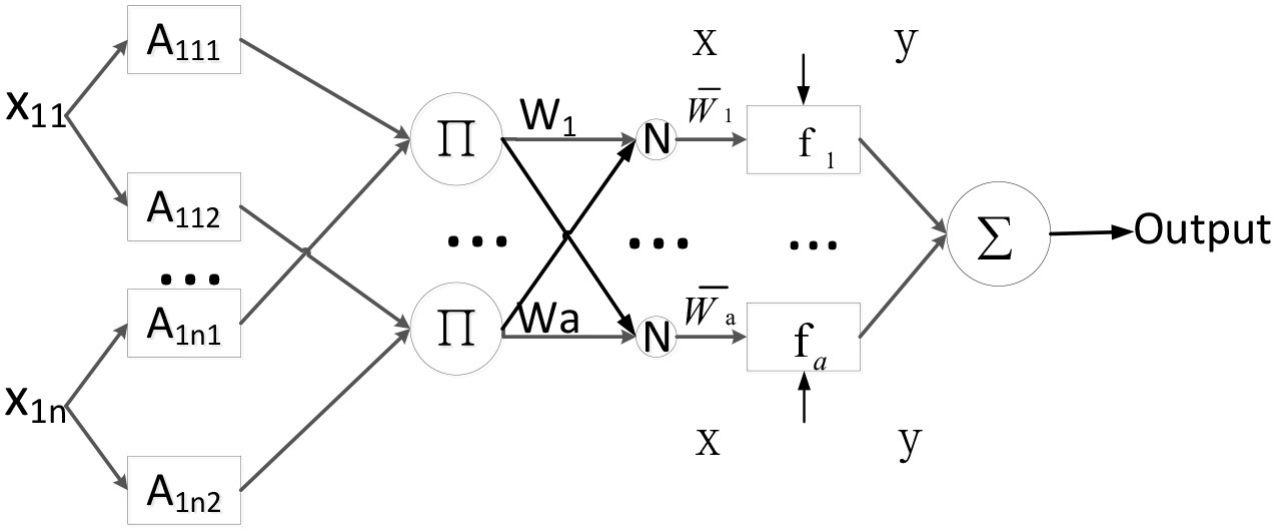
Architecture of ANFIS.

ANFIS algorithm [38] employs a fuzzy inference module based on empirical knowledge to make the final decision. The basic structure of most fuzzy inference systems (FISs) is a model that maps the input characteristics to the input membership functions (MF). ANFIS is a multilayer feedforward network consisting of five layers. Each layer in the ANFIS architecture contains nodes defined by node functions, as shown in Fig 4.

The five layers of ANIFIS are:

**Layer1**: Calculate the membership grade of the inputs with the membership function (MF). In this study, we used Gaussian MF.
$$\mu_{A_{1nj}}(x_{1n})=e^{-{\left(\frac{x-mean_{n}}{sigma_{n}}\right)}^{2}}$$ (2)
  Where *x*_1*n*_ is the input to the node *A*1_*n*_. *n* is the number of attributes. *j* is the linguistic label (small, large, etc.) of attribute *n*. *A*_1*nj*_ is the membership that determines the degree to which input *x*_1*n*_ satisfies *A*_1*nj*_. *mean*_*n*_ represents the center of the Gaussian function. *sigma*_*n*_ represents the width of the Gaussian function. *mean*_*n*_ and *sigma*_*n*_ are the learning parameters. They are referred to as premise parameters. As the values of these parameters change, the Gaussian functions vary accordingly.
**Layer2**: Firing strengths. Each node in this layer calculates the firing strength of the *i*-th rule.
$$w_{i}=\mu_{A_{11j}}(x_{11})\times \ldots \times \mu_{A_{1nj}}(x_{1n})$$ (3)
  Where *i* is the node number of Layer2. Each node output represents the firing strength of a rule.
**Layer3**: Calculate the normalized firing strength of the *i*-th rule.
$${\bar{w}}_{i}=\frac{w_{i}}{\sum_{i=1}^{a}w_{i}}$$ (4)
  Where *a* is the number of nodes in Layer2.
**Layer4**: Adaptive node with a linear function. Each node calculates the weighted value of the consequent part of each rule.
$${\bar{w}}_{i}f_{a}={\bar{w}}_{i}(p_{i}x+q_{i}y+r_{i})$$ (5)
  Where *p*_*i*_, *q*_*i*_, *r*_*i*_ are the learning parameters. These parameters are referred to as consequent parameters.
**Layer5**: Produce the overall output by aggregating all the fired rule values.
$$output=\sum_{i}{\bar{w}}_{i}\cdot f_{i}$$ (6)
ANFIS uses a hybrid learning mechanism to train the model. The main learning parameters of ANFIS are premise parameters and consequent parameters. In the forward-passing process of hybrid learning, the node output is propagated to the fourth layer, and the least-squares method is used to estimate the consequent parameters. In backward propagation, the loss (the difference between the expected output and the actual output) is propagated back to the first layer, with the premise parameters updated using gradient descent, while the consequent parameters are fixed.

### Deep residual Network-ResNet

ResNet is one of CNN’s variants. It is a large-scale convolutional neural network constructed by residual blocks, 20 times larger than AlexNet [39] and 8 times larger than VGG-16 [40]. It excels in image recognition and is the winner of the image classification and object recognition algorithms in the 2015 Image Net Large Scale Visual Recognition Competition. It also outperforms the third version of GoogLeNet [41].

**Fig 5:**
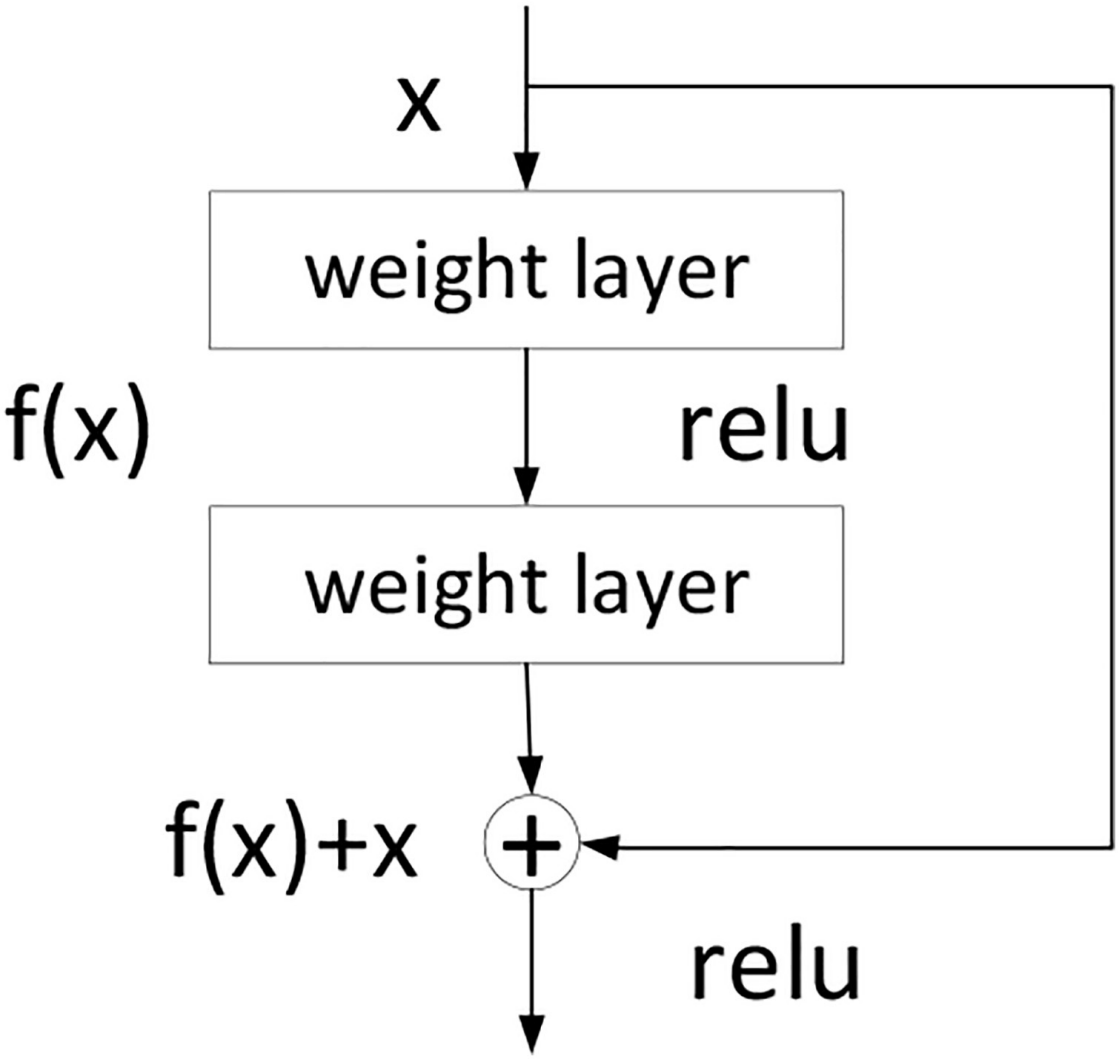
Residual block of ResNet.

ResNet comes from an artificial intelligence team of Microsoft [42]. Because of the residual mechanism, the depth of the network can be deeper than that of the traditional networks, which can effectively avoid the problems of gradient disappearance and training difficulties of the deep network. The structure of the residual block is shown in Fig 5.

In Fig 5, *x* is the input of a residual block; *f*(*x*) is the output of the residual block before the second activation function. That is to say, *f*(*x*) = *W*_2_*σ*(*W*_1_*x*), where *W*_1_ and *W*_2_ are the weights of the first and second layers, and *σ* is the rectified linear unit (ReLU) activation function. The output of the residual block is *σ*(*f*(*x*) + *x*).

**Fig 6:**
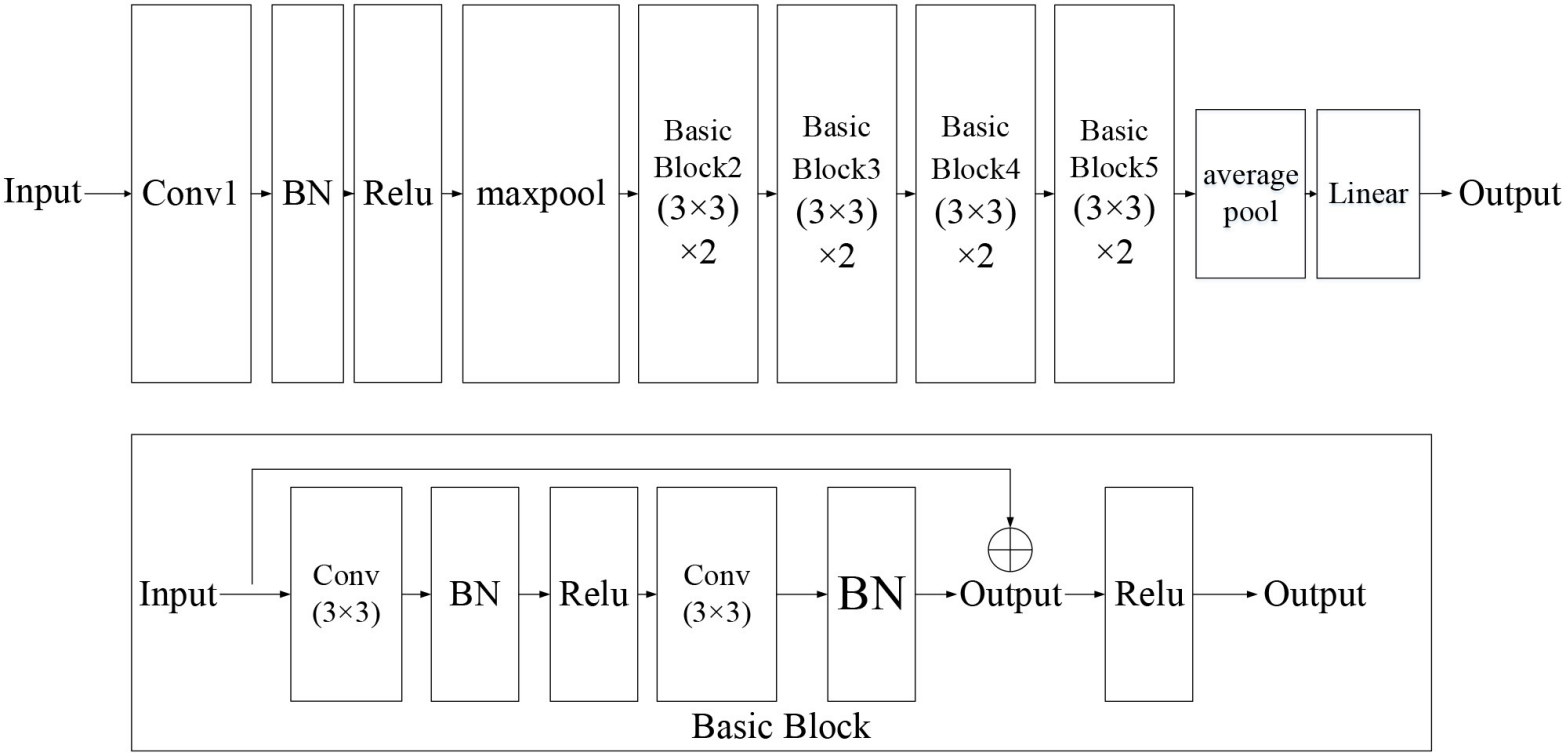
The structure of ResNet18.

In the original paper proposed the ResNet, five different architectures are presented, including 18-layer, 34-layer, 50-layer, 101-layer, and 152-layer ResNet. The more layers, the more time it takes to train and predict. Fig 6 shows the network structure of ResNet18. There are 8 basic block modules in ResNet18. The arrow in the basic block is the shortcut connection. These shortcut connections allow the neural network to be deeper because gradients can transfer farther in the backpropagation process.

## Proposed methodology

In this section, we first demonstrate the preprocessing techniques in the proposed method, including the attributes selection method and the techniques of initializing the overlapped intervals of continuous attributes. Then, the proposed method is explained detailed.

### Preprocessing

First, we conducted correlation analysis and attribute selection for the data. Since the Layer2 of ANFIS uses the multiplication of all incoming signals to form rules, a large number of rules are generated, as shown in Eq (3). Therefore, it is necessary to conduct preliminary attribute selection for problems with many attributes. The purpose of correlation analysis is to select the continuous attributes, which will be the input of the proposed method’s ANFIS-part.

In this study, we used Pearson correlation analysis to select continuous attributes, which will be the input of the proposed method’s ANFIS-part. And we use 0.1 as the minimum threshold for selecting attributes. An important advantage of using Pearson correlation analysis to select continuous attributes is that the selected continuous attributes have a higher degree of linear correlation with the target.

Second, the standard deviation is used to determine the original interval number of continuous attributes selected by Pearson correlation analysis. Standard deviation reflects the degree of dispersion of an attribute. The larger the standard deviation is, the more intervals are needed to represent the dispersion. Therefore, in this study, we related the original number of attribute intervals to the exponent of the standard deviation. We used the exponent of the standard deviation of each selected continuous attribute plus 2 as the original interval number.

**Fig 7:**
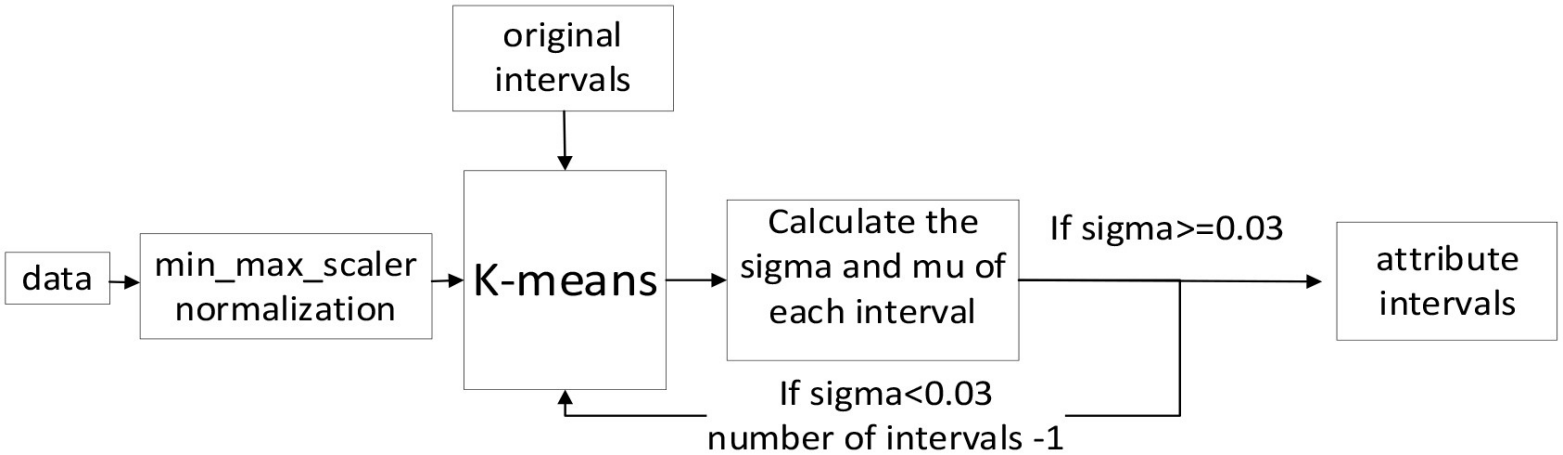
Adaptive K-means algorithm.

Third, to further minimize and check the number of intervals, we proposed an adaptive K-means to dynamically determine the number of intervals. This is because although K-means is one of the most popular clustering algorithms [37], it also has some disadvantages, one of them is the number of clusters *k* of K-means must be known in advance. To dynamically determine the minimum number of clusters *k*. An adaptive K-means algorithm is proposed (see Fig 7).

The adaptive K-means algorithm is based on the following four stages:

**Stage 1** Min_max_scaler normalization is used to normalize all attributes. This is to eliminate the dimensional influence among attributes.
**Stage 2** K-means is used to calculate the cluster centers of each interval. The original interval number of K-means is calculated from the standard deviation, which is the exponent of the standard deviation of each attribute plus 2.
**Stage 3** Divide the data into intervals and calculate the *sigma* and *mean* of each interval as below. If one interval’s *sigma* is less than 0.03 which means insufficient dispersion of data on this interval, the clustering number will be reduced by 1, and the clustering is returned to Stage2 for re-clustering.
$$mean=\frac{\sum_{1}^{n}x_{i}}{n}$$ (7)
$$sigma=\sqrt{\frac{\sum_{1}^{n}{\left(x_{i}-mean\right)}^{2}}{n}}$$ (8)
  Where *n* is the total amount of data.
**Stage 4** If the *sigma* of each attribute is greater than 0.03, the attribute is finally distributed according to the clustering intervals. And the calculated *sigma* and *mean* of intervals will be the original *sigma* and *mean* before being trained by the proposed method, which will make the *sigma* and *mean* of the ANFIS at an appropriate level during the initial phase.

### Connection of ANFIS and ResNet

**Fig 8:**
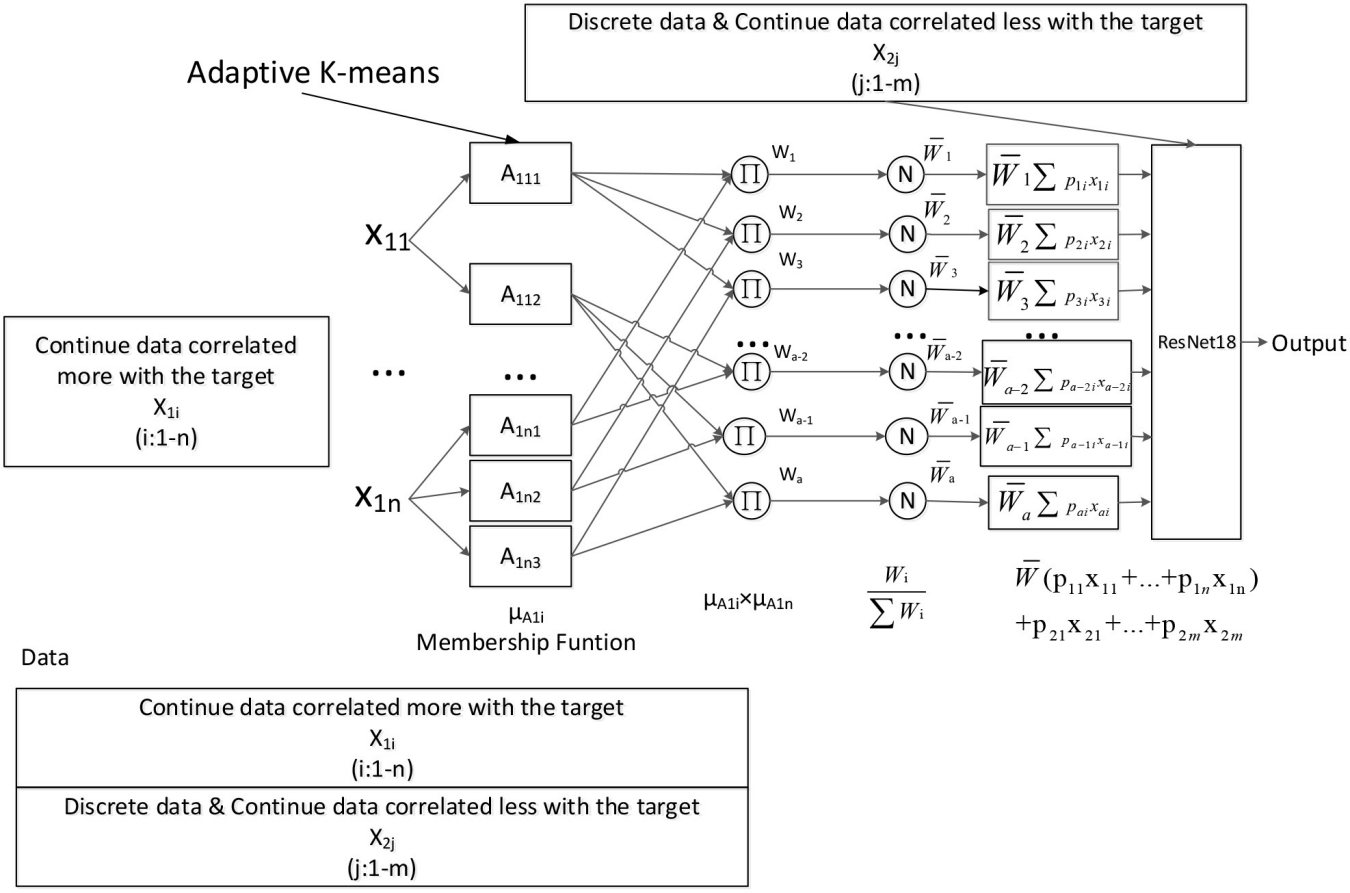
The architecture of the proposed method.

In the proposed method, ANFIS is used to generate the overlapped continuous attributes’ intervals and fuzzy rules (fuzzy combinations of selected continuous attributes), and ResNet is used to identify the deep features in the generated fuzzy rules and discrete attributes. For this purpose, we connect the modified ANFIS algorithm to the structure of ResNet for deep pattern extraction and co-train the two algorithms, as shown in Fig 8.

The layers in the proposed method are demonstrated below:

**Layer1**: According to the *means* and *sigmas* calculated by adaptive K-means to form the Gaussian membership functions (MFs). Then, these MFs are used to calculate the membership grade of the inputs.
$$\mu_{A_{1nj}}(x_{1n})=e^{-{\left(\frac{x-mean_{n}}{sigma_{n}}\right)}^{2}}$$ (9)
  Where *mean* and *sigma* are the learning parameters.
**Layer2**: Firing strengths. Each node in this layer calculates the firing strength of the *i*-th rule.
$$w_{i}=\mu_{A_{11j}}(x_{11})\times \ldots \times \mu_{A_{1nj}}(x_{1n})$$ (10)
**Layer3**: Calculate the normalized firing strength of the *i*-th rule.
$${\bar{w}}_{i}=\frac{w_{i}}{\sum_{i=1}^{a}w_{i}}$$ (11)
  Where *a* is the number of rules.
**Layer4**: $WF=\left\{WF_{1},WF_{2},\cdots ,WF_{a}\right\},\;WF_{i}=\left\{\bar{w_{i}}f_{1},\bar{w_{i}}f_{2},\cdots ,\bar{w_{i}}f_{d}\right\}$. Weighted rules set.
$${\bar{w}}_{i}f_{con\_sel\_i}={\bar{w}}_{i}p_{con\_sel\_i}x_{con\_sel\_i}$$ (12)
  Where *p*_*con*_*sel*_*i*_ is the learning parameter.
**Layer5**: Concatenate the weighted rules, discrete date, and the other continuous data as inputs of ResNet18.
$$X^{\prime }=\text{WF}+Q_{1}X_{dis}+Q_{2}X_{con\_other}+R$$ (13)
**Layer6**: Calculate the result *Y*′ by ResNet18. Then, the algorithm calculates and feedback the gradient to co-train the learning parameters, including the normal learning parameters of ResNet and the *mean*, *sigma* and *p*_*con*_*sel*_*i*_ parameters of ANFIS, as shown in Eqs (9) and (12). The actual algorithm is shown in Algorithm 1.

**Algorithm 1** The proposed algorithm

**Input**: $\left(X_{con\_sel},X_{dis},X_{con\_other}\right)\in {\mathbb{R}}^{m\times d},\;X_{con\_sel}\in {\mathbb{R}}^{m\times d_{1}}$ is selected continuous data, $X_{dis}\in {\mathbb{R}}^{m\times d_{2}}$ is discrete data, $X_{con\_other}\in {\mathbb{R}}^{m\times d_{3}}$ is other continuous data. *d* = *d*_1_ + *d*_2_ + *d*_3_.

**Output**: Classification result $Y\in {\mathbb{R}}^{m}$.

Step 1. ***ANFIS***(*X*_*con*_*sel*_) → (*WF*):

- The original Gaussian membership functions (formed by *means* and *sigmas* after Adaptive K-means).
$$\mu (x)=e^{-\frac{1}{2}{\left(\frac{x-mean}{sigma}\right)}^{2}}$$ (14)
- *w*_*i*_, firing strengths of *i*-th rule, as follows:
$$w_{i}=\mu_{A_{11j}}(x_{11})\times \ldots \times \mu_{A_{1nj}}(x_{1n})$$ (15)
- $\bar{w_{i}}$, firing strengths of *i*-th rule, as follows:
$${\bar{w}}_{i}=\frac{w_{i}}{\sum_{i=1}^{a}w_{i}}$$ (16)
  Where *a* is the number of rules.
- $WF=\left\{WF_{1},WF_{2},\cdots ,WF_{a}\right\},\;WF_{i}=\left\{\bar{w_{i}}f_{1},\bar{w_{i}}f_{2},\cdots ,\bar{w_{i}}f_{d}\right\}$, weighted rules set, each weighted rule can be calculated as follows:
$${\bar{w}}_{i}f_{con\_sel\_i}={\bar{w}}_{i}p_{con\_sel\_i}x_{con\_sel\_i}$$ (17)

Step 2. ***ResNet***(*WF*, *X*_*dis*_, *X*_con_other_)→(*Y*′):

- Concatenate *WF*, *X*_*dis*_, *and* *X*_*con*_*other*_ as:
$$X^{\prime }=WF+Q_{1}X_{dis}+Q_{2}X_{con\_other}+R$$ (18)
  Where $Q_{1}=\left\{q_{11},q_{12},\cdots ,q_{1d_{2}}\right\}\in {\mathbb{R}}^{m\times d_{2}}$, $Q_{2}=\left\{q_{21},q_{22},\cdots ,q_{2d_{3}}\right\}\in {\mathbb{R}}^{m\times d_{3}}$, $R=\left\{r_{1},r_{2},\cdots ,r_{m}\right\}\in {\mathbb{R}}^{m\times 1}$.
- Calculate ***ResNet*18**(*X*′) to get *Y*′. The structure of ResNet18 is shown in Fig 6.

Step 3. ***Backpropagation***(*Y*′, *Y*):

- Adam optimizer[43] to transfer the gradient to Layer5. Then the gradient divides into two parts:
  The *Q*_*1*_ and *Q*_*2*_ are trained by Adam (learning rate = 0.001)
  the *W* is trained by SGD (learning rate = 1e-4, momentum = 0.99)
- Pass the gradient to Layer1. Use the hybrid train to train the *sigmas* and *means*.

Step 4. ***Verify***_***Loss***(*Y*′, *Y*):

- Calculate the loss of (*Y*′, *Y*) on each epoch.
- Early Stopping mechanism (patience = 5). If the loss keeps no improvement after 5 epochs, stop training.

In the training process, the intervals of the selected continuous attribute *x*_1*i*_ are divided according to the standard deviation and adaptive K-means, which will generate overlapped intervals. Then, the membership function is used to turn the value of these continuous attributes into the membership degree belonging to the intervals. By not just providing the exact continuous value, but the membership degree of overlapped intervals (e.g., big, medium, small), these membership degrees will provide certain fuzziness property for the proposed algorithm. Then, fuzzy rules (product of different interval membership degrees of attributes) are generated to represent different possible combinations of continuous selected attributes (such as duration is small, the count is medium, diff_srv_rate is big). After the normalization of all the *W*_*i*_ (rules’ firing strengths), weighted fuzzy rules are formed, which represent the rules’ contribution to the target. Finally, the weighted fuzzy rules, discrete data, and the remaining continuous attributes will be concatenated to input ResNet. Compared with the original ResNet, the ANFIS-enhanced ResNet will transfer the exact continuous value to fuzzy and overlapped intervals’ membership degree, generate fuzzy rules (various fuzzy combinations of selected continuous attributes), and provide more fuzzy characteristics for ResNet.

## Results and discussion

The simulation was carried out using Python 3.7, PyTorch, and sklearn on the NSL-KDD dataset. And the performance analysis and comparison with other studies are conducted in this section.

### Data description

NSL-KDD (National security lab–knowledge discovery and data mining) is a traditional benchmark dataset in intrusion detection. It is the enhanced form of the KDD99 dataset to outperform its limitations. It is a public dataset, which can be downloaded from https://www.unb.ca/cic/datasets/nsl.html. The NSL-KDD dataset has total of 43 attributes, including 41 normal attributes, 1 target attribute, and 1 hard attribute (hard attribute is used to describe the hard degree of classification). Of the 41 attributes, 7 are discrete and 34 are continuous.

In the NSL-KDD dataset, the most commonly used subset of NSL-KDD is the KDDTrain+ subset and the KDDTest+ subset. The NSL-KDD dataset can test the generalization ability of an algorithm because the traffic distribution of the KDDTrain+ subset and KDDTest+ subset is different. Some new intrusion types exist only in the KDDTest+ subset. The KDDTrain+ subset of the NSL-KDD dataset contains 23 classes, including 22 types of attacks and normal. The KDDTest+ subset of the NSL-KDD dataset contains 38 classes, including 37 types of attacks and normal. The actual traffic distribution of KDDTrain+ and KDDTest+ is shown in Table 1.

**Table 1:** Distribution of five major categories behaviours of KDDTrain+ and KDDTest+.

Category	KDDTrain+ dataset		KDDTest+	
	Count	Percentage	Count	Percentage
Normal	67342	53.46%	9711	43.08%
Probe	45927	36.46%	7457	33.08%
DOS	11656	9.25%	2754	12.22%
U2R	995	0.79%	2421	10.74%
R2L	52	0.04%	200	0.89%
Total	125972	100%	22543	100%

In this study, an 80% KDDTrain+ subset was used as the training dataset. The full KDDTrain+ subset and KDDTest+ subset served as the validation and test set, respectively.

### Performance metrics

In intrusion detection, samples containing attacks can be defined as positive samples, and samples without attacks can be defined as negative samples. The results of the intrusion detection can be divided into the following four situations: TP (True Positive), TN (True Negative), FP (False Positive), and FN (False Negative), as shown in Table 2. The proposed method will be evaluated by precision, recall rate, F1-Score, ACC (accuracy), DR (detection rate), and FAR (false alarm rate). These metrics can be calculated by TP, TN, FP, and FN.

**Table 2:** Four situations of results.

Classification	Positive	Negative
Sample		
Positive	True Positive (TP)	False Negative (FN)
Negative	False Positive (FP)	True Negative (TN)

Precision: The percentage of all TP samples to all positive classifications (TP+FP).


$$\text{Pr}ecision=\frac{TP}{TP+FP}$$ (19)

Recall rate: The percentage of all TP samples to all samples (TP+FN) that should be positive.


$$\text{Re}call=\frac{TP}{TP+FN}$$ (20)

Accuracy (ACC): The percentage of all correct predictions.


$$ACC=\frac{TP+TN}{TP+FN+FP+FN}$$ (21)

F1-Score: Harmonic mean of precision and recall rate. A high F1-Score indicates high precision and recall. When the precision and recall are both 100%, the best F1-Score is 1. The worst F1-Score is 0.


$$F_{1}=2\times \frac{\text{Pr}ecision\times \text{Re}call}{\text{Pr}ecision+\text{Re}call}$$ (22)

DR (Detection Rate): The percentage of successfully categorizing this data in this category.


$$DR=\frac{TP}{TP+FN}$$ (23)

FAR (False Alarm Rate): The percentage of data that should not be identified for this category.


$$FAR=\frac{FP}{TN+FP}$$ (24)

### Experimental results

We evaluated the proposed method with the NSL-KDD dataset. 80% KDDTrain+ subset was used as the training dataset. The full KDDTrain+ subset and KDDTest+ subset served as the validation and test set, respectively. S2 Table shows the results of Pearson correlation analysis, which is the Pearson correlation degree of 41 normal attributes towards the target attribute. In order to select the continuous attributes which are more related to the target, we take the correlation degree’s absolute value greater than 0.1 as the threshold and select 18 continuous attributes, as shown in Table 3, which reduces the dimension of data in the ANFIS-part and the number of generated rules. And the total Pearson correlation matrix figure of the NSL-KDD dataset’s 43 attributes is shown in S1 Fig.

**Table 3:** The standard deviation and original interval number of attributes.

Attribute Name	Standard Deviation	Original Interval Number
dst_host_srv_diff_host_rate	1.13E-01	1
diff_srv_rate	1.80E-01	1
dst_host_diff_srv_rate	1.89E-01	1
dst_host_rerror_rate	3.07E-01	1
dst_host_same_src_port_rate	3.09E-01	1
dst_host_srv_rerror_rate	3.19E-01	1
rerror_rate	3.20E-01	1
srv_rerror_rate	3.24E-01	1
same_srv_rate	4.40E-01	1
dst_host_serror_rate	4.45E-01	1
dst_host_srv_serror_rate	4.46E-01	1
serror_rate	4.46E-01	1
srv_serror_rate	4.47E-01	1
dst_host_same_srv_rate	4.49E-01	1
dst_host_count	9.92E+01	3
dst_host_srv_count	1.11E+02	4
count	1.15E+02	4
duration	2.60E+03	5

In order to minimize the number of selected continuous attributes’ intervals and initialize the *sigma* and *mean* of each interval, first, through the standard deviation analysis, the original number of selected continuous attributes’ intervals is shown in Table 3. And, after the proposed adaptive K-means algorithm, the final interval number, *sigmas* and *means* of each selected continuous attribute are shown in Table 4. In fact, the number of fuzzy rules generated by the ANFIS-part is reduced to 192, as shown in the S1 Table.

**Table 4:** Final interval number of selected attributes.

Attribute Name	Final Interval Number	Original mean and Sigma (First is mean, second is sigma)
diff_srv_rate	1	[0.6, 1.8]
dst_host_diff_srv_rate	1	[0.8, 1.9]
dst_host_rerror_rate	1	[1.2, 3.1]
dst_host_same_src_port_rate	1	[1.5, 3.1]
dst_host_same_srv_rate	1	[5.2, 4.5]
dst_host_serror_rate	1	[2.8, 4.4]
dst_host_srv_diff_host_rate	1	[0.3, 1.1]
dst_host_srv_rerror_rate	1	[1.2, 3.2]
dst_host_srv_serror_rate	1	[2.8, 4.5]
rerror_rate	1	[1.2, 3.2]
same_srv_rate	1	[6.6, 4.4]
serror_rate	1	[2.8, 4.5]
srv_rerror_rate	1	[1.2, 3.2]
srv_serror_rate	1	[2.8, 4.5]
dst_host_count	3	[1.0, 0.9], [5.1, 1.3], [9.9, 0.4]
dst_host_srv_count	4	[0.4, 0.4], [2.9, 0.8], [6.2, 1.0], [9.9, 0.4]
count	4	[0.2, 0.2], [2.4, 0.6], [4.8, 0.7], [9.5, 0.8]
duration	4	[0.0, 0.1], [2.1, 0.7], [4.9, 0.9], [8.7, 0.9]

**Fig 9:**
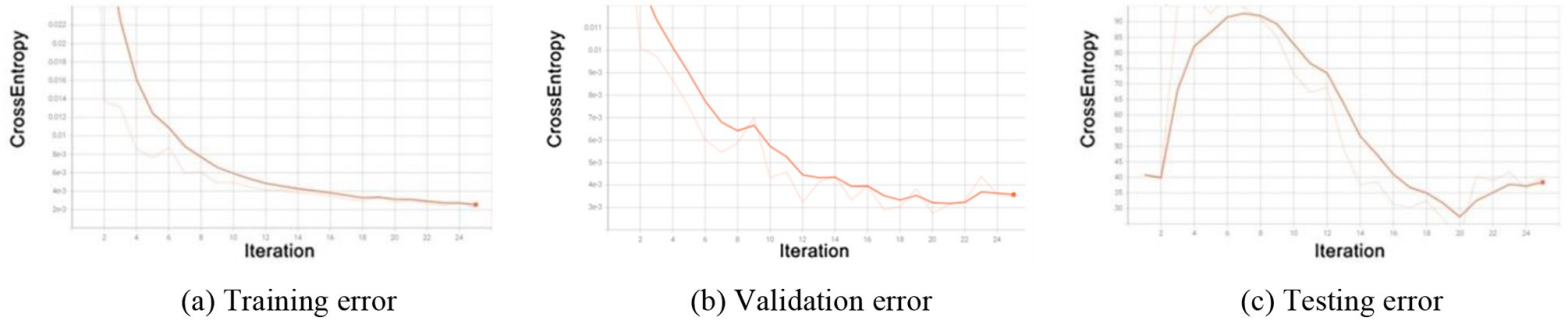
CrossEntropy convergence curve of the proposed method.

Then, through Algorithm 1, the ANFIS-part of the proposed method generated fuzzy rules (fuzzy combinations of selected continuous attributes), and the ResNet-part of the proposed method identified the deep pattern in the fuzzy rules and discrete data. The whole architecture is co-trained with the loss (the difference between the actual *Y* and the predicted *Y*′). After this process, the post-training ANFIS-part rules with four main continuous attributes (dst_host_count, dst_host_srv_count, count, duration) are shown in the S1 Table. The training, validation, and testing error changes in this process are shown in Fig 9. We can see that in the initial training phase, the model presents an overfitting problem. However, after 8 epochs, the overfitting problem begins to ease and keeps a good performance.

The performance of the proposed method on different metrics is shown in Tables 5 and 6. According to the experimental result on the KDDTrain+ dataset, as shown in Table 5, the method presents a high detection rate and low false alarm rate. The DR on the KDDTrain+ dataset reaches 98.88%. And the FAR on KDDTrain+ dataset is only 1.11%. On the KDDTest+ dataset (some intrusions only exist in the KDDTest+ dataset), the proposed method performs a certain generalization ability. The DR on the Normal category reaches 96.67%. The Precision on the DOS category reaches 93.14%. And, overall, the method reaches a total of 75.9% DR, 79.42% Precision, and 75.91% ACC on the KDDTest+ dataset. However, the FAR on the KDDTest+ dataset is not ideal, accounting for 24.15%, which needs to be further improved.

**Table 5:** Experimental results for different performance metrics in five categories of the KDDTrain+ dataset.

Category	Precision (%)	Recall (%)	F1-score(%)	DR(%)	FAR(%)	ACC(%)
Normal	99.11	98.91	99.01	98.91	0.89	99.01
DOS	99.61	99.88	99.74	99.88	0.39	99.74
Probing	95.32	96.89	96.10	96.89	4.76	96.07
R2L	91.78	77.39	83.97	77.39	6.93	85.23
U2R	100	46.15	63.16	46.15	0.00	73.08
weighted avg	98.88	98.89	98.87	98.88	1.11	98.88

**Table 6:** Experimental results for different performance metrics in five categories of the KDDTest+ dataset.

Category	Precision (%)	Recall (%)	F1-score(%)	DR(%)	FAR(%)	ACC(%)
Normal	68.88	96.67	80.44	96.67	43.68	76.50
DOS	93.14	79.92	86.03	79.92	5.89	87.02
Probing	68.69	65.96	67.30	65.96	30.07	67.95
R2L	88.83	5.77	10.84	5.77	0.73	52.52
U2R	80.00	4.00	7.62	4.00	7.62	51.50
weighted avg	79.42	75.91	71.73	75.90	24.15	75.91

The performance of the proposed method was also compared with other studies using the same dataset, such as MVO-ANN [22], FC-ANN [36], DNN [44], CSO-ANFIS [36], and original ResNet. In terms of the classification performance of five main categories, the comparison of the performance with these classifiers is shown in Tables 7 and 8.

**Table 7:** Detection rate, ACC, and FAR comparison of different classifiers on KDDTrain+.

Classifier	DR	FAR	ACC	F1-score	Recall	Precision
MVO-ANN [22]	96.25	**0.032**	98.21			
FC-ANN [36]	89.23	4.37				
DNN [44]			91.7			
CSO-ANFIS [36]	95.80	3.45				
DBN+ResNet101 [45]			**98.95**	97.62	98.76	**98.94**
ResNet	43.42	56.58	43.42			
Proposed Method	**98.88**	1.11	98.88	**98.87**	**98.89**	98.88

**Table 8:** ACC comparison of different classifiers on KDDTrain+.

Classifier	Normal	DOS	Probing	R2L	U2R
BPNN [36]	91.50	90.94	80.53	48.13	60.00
GA-DBN [14]		99.45	97.78	**99.37**	**98.68**
FC-ANN [36]	93.87	80.32	89.12	89.57	75.58
GA-ANFIS [36]	96.22	96.70	93.18	94.89	83.33
PSO-ANFIS [36]	96.46	95.90	91.35	94.72	85.62
CSO-ANFIS [36]	97.41	96.25	92.51	94.15	90.26
ResNet	53.66	38.69	3.53	35.48	50.00
Proposed Method	**99.87**	**99.92**	**99.79**	97.79	80.77

According to the comparison, the detection rate of the proposed method is 55.46% better than that of the original ResNet. The false alarm rate is 55.47% less than ResNet. This means that the proposed method in this paper alleviates the overfitting problem of ResNet to some extent. Also, the performance on various metrics is improved and is better than many other methods. Compared with other methods, the DR on the KDDTrain+ dataset is the best, 2.63% higher than MVO-ANN [22]. The FAR is only worse than MVO-ANN[22]. The F1-score is 1.25% higher than DBN+ResNet101 [45]. The recall is 0.13% higher than DBN+ResNet101 [45]. The ACC is only 0.07% lower than DBN+ResNet101 [45]. The Precision is only 0.06% lower than DBN+ResNet101 [45]. And, except for U2R and U2R, in the other three main categories, the ACC is all superior to CSO-ANFIS, BPNN, GA-DBN, etc.

## Conclusion

There are two important experiments conducted in the study. The first one is the ANFIS-enhanced ResNet structure. In this experiment, the concatenation of modified ANFIS and ResNet generates a fuzzy-enhanced architecture to improve the fuzzy ability of ResNet. The second one is the efficiency improvement of the proposed algorithm. In the experiment, we used Pearson correlation analysis to select 18 continuous attributes that are highly related to the target as the inputs of the proposed method’s ANFIS-part. And, for determining the meaningful and minimum interval division of ANFIS-part, standard deviation and proposed adaptive K-means algorithm are used to dynamically determine the original interval division of each selected continuous attribute.

The performance of the proposed method was compared with other algorithms: BPNN, GA-DBN, DNN, FC-ANN, GA-ANFIS, PSO-ANFIS, CSO-ANFIS, and original ResNet. The proposed method achieved a 98.88% detection rate and 1.11% false alarm rate on the KDDTrain+ dataset, which is better than these methods on various metrics. Meanwhile, compared with the original ResNet, the performance on various metrics has been improved, which presents an enhanced generalization ability.

## Supporting information

S1 Fig Correlation matrix between attributes of NSL-KDD dataset. (PNG)

S1 Table The generated rules with four attributes of modified ANFIS. (DOCX)

S2 Table Correlation degree of 41 attributes towards target. (DOCX)
