# Supplementary figures and images for: On the combination of adaptive neuro-fuzzy inference system and deep residual network for improving detection rates on intrusion detection
Source: PLoS One. 2022 Dec 12;17(12):e0278819. doi: 10.1371/journal.pone.0278819 (PMC9744302; doi:10.1371/journal.pone.0278819)

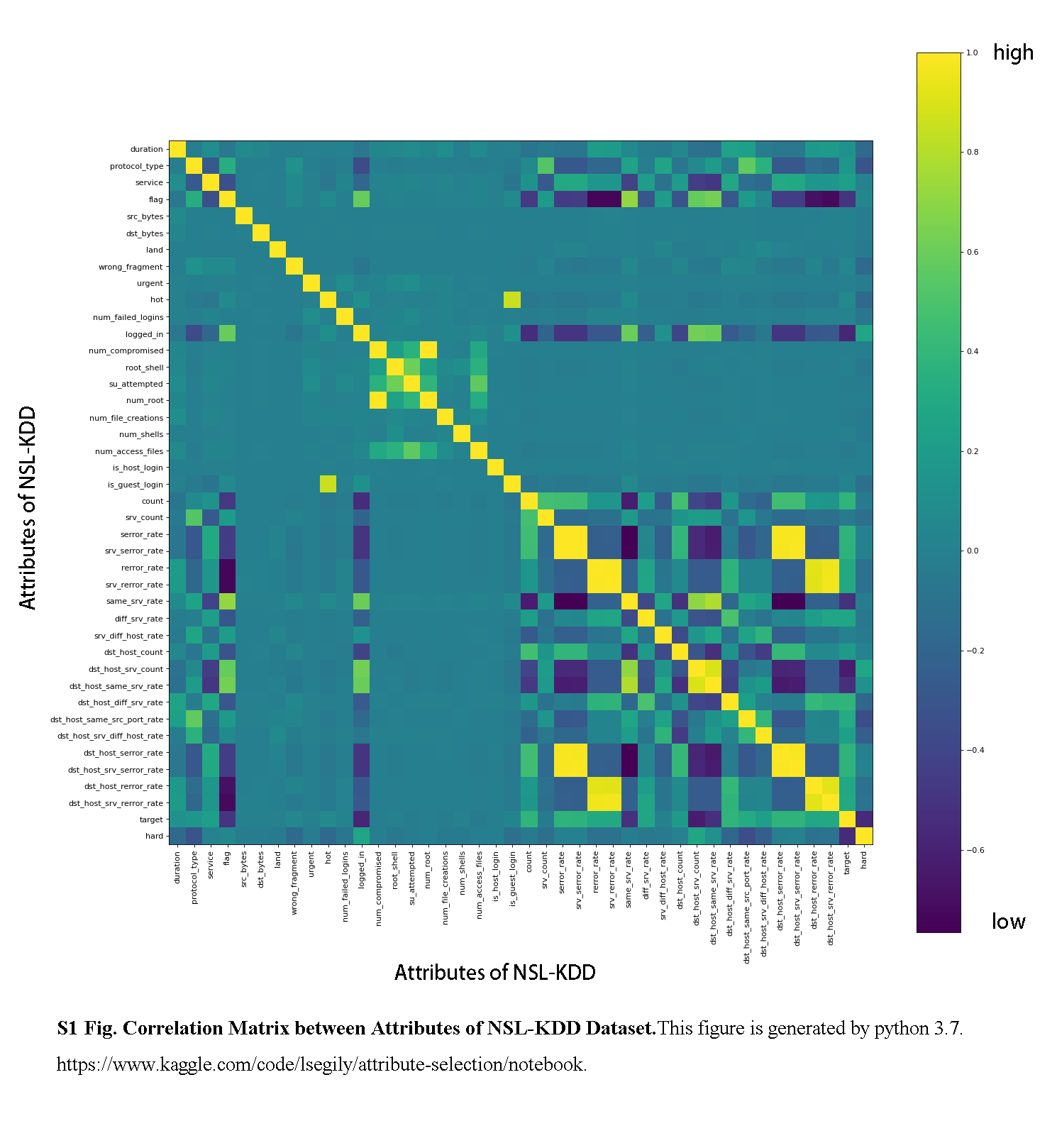

Supplement: S1 Fig — (PNG) [file pone.0278819.s001.png]
