# Supplementary material for: On the combination of adaptive neuro-fuzzy inference system and deep residual network for improving detection rates on intrusion detection
Source: PLoS One. 2022 Dec 12;17(12):e0278819. doi: 10.1371/journal.pone.0278819 (PMC9744302; doi:10.1371/journal.pone.0278819)
Supplement: S1 Table — (DOCX) [file pone.0278819.s002.docx]

**S1 Table. The Generated Rules with Four Attributes of Modified ANFIS**

| rule number | duration | count | dst_host_count | dst_host_srv_count | r |
| --- | --- | --- | --- | --- | --- |
| rule0 | 0.912633 | 0.038782 | 0.382223 | 0.188299 | 0.246868 |
| rule1 | 0.793117 | 0.387096 | 0.576429 | 0.048162 | 0.698428 |
| rule2 | 0.324191 | 0.604768 | 0.496349 | 0.227386 | 0.157725 |
| rule3 | 0.2888 | 0.002117 | 0.045971 | 0.40616 | 0.429863 |
| rule4 | -0.17317 | 0.474115 | 0.173647 | 0.405452 | 0.196219 |
| rule5 | 0.992505 | 0.924066 | 0.558316 | 0.100145 | 0.120558 |
| rule6 | -0.10636 | 0.439569 | 0.128769 | 0.715641 | 0.467034 |
| rule7 | 0.544545 | 0.61816 | 0.716447 | 0.048606 | 0.92977 |
| rule8 | -0.46834 | 0.692778 | 0.723793 | 0.561677 | 0.796844 |
| rule9 | 0.39182 | 0.44647 | 0.795715 | 0.733722 | 0.600022 |
| rule10 | 0.023456 | 0.022299 | 0.118969 | 0.748522 | 0.753872 |
| rule11 | 0.766467 | 0.596883 | 0.069285 | 0.094784 | 0.123723 |
| rule12 | 0.844623 | 0.314823 | 0.709759 | 0.727116 | 0.61416 |
| rule13 | 0.326573 | 0.558993 | 0.743943 | 0.866395 | 0.100346 |
| rule14 | 0.940911 | 0.744211 | 0.446172 | 0.212663 | 0.180843 |
| rule15 | 0.903323 | 0.483978 | 0.457087 | 0.174246 | 0.384277 |
| rule16 | 0.344584 | 1.457618 | 0.720129 | 1.056152 | 0.971608 |
| rule17 | 0.386902 | 0.608888 | 0.465949 | 0.720046 | 0.455176 |
| rule18 | -0.01638 | 0.180129 | 0.446932 | 0.711239 | 0.882801 |
| rule19 | 0.286007 | 0.900344 | 0.809844 | 0.465056 | 0.335725 |
| rule20 | 0.132107 | 0.310317 | 0.257752 | 0.945694 | 0.764025 |
| rule21 | 0.990796 | 0.337944 | 0.953611 | 0.740688 | 0.192604 |
| rule22 | 0.81035 | 0.931257 | -0.00323 | 0.488747 | 0.276794 |
| rule23 | 0.269117 | 0.249037 | 0.799538 | 0.094236 | 0.044439 |
| rule24 | 0.25111 | 0.863521 | 0.877058 | 1.125156 | 1.137547 |
| rule25 | 0.782634 | 0.474079 | 0.593925 | 0.607624 | 0.419111 |
| rule26 | 0.857426 | 0.384196 | 0.335954 | 0.914791 | 0.184694 |
| rule27 | 0.270526 | 0.719492 | 0.526085 | 0.125003 | 0.222262 |
| rule28 | 0.900054 | 0.430908 | 0.656443 | 0.297443 | 1.1813 |
| rule29 | 0.592964 | 0.393583 | 0.202679 | 0.275164 | 0.24013 |
| rule30 | 0.320082 | 0.683497 | 0.496568 | 0.54302 | 0.680666 |
| rule31 | 0.068587 | 0.104896 | 0.587402 | 0.160419 | 0.736977 |
| rule32 | 0.958706 | 0.397265 | 0.144433 | 0.640488 | 0.616761 |
| rule33 | 0.399153 | 0.611011 | 0.596781 | 0.490967 | 0.347192 |
| rule34 | 0.139596 | 0.550007 | 0.831538 | 0.120628 | 0.742352 |
| rule35 | 0.832145 | 0.661792 | 0.142331 | 0.627675 | 0.82275 |
| rule36 | 0.82718 | 0.392866 | 0.267327 | 0.084517 | 0.507285 |
| rule37 | 0.475409 | 0.575311 | 0.395607 | 0.281335 | 0.866238 |
| rule38 | 0.468848 | 0.89154 | 0.026081 | 0.656689 | -0.00754 |
| rule39 | 0.685748 | 0.627479 | 0.416181 | 0.392638 | 0.415244 |
| rule40 | 0.998556 | 0.827017 | 0.021562 | 0.706549 | 0.592641 |
| rule41 | 0.956073 | 0.55833 | 0.918837 | 0.442208 | 0.662319 |
| rule42 | 0.210139 | 0.429479 | 0.650655 | -0.01814 | 0.643103 |
| rule43 | 0.326526 | 0.368585 | 1.151617 | 0.534994 | 0.283283 |
| rule44 | 0.17348 | 0.651012 | 0.545738 | 0.293967 | 0.888717 |
| rule45 | 0.060504 | 0.742123 | 0.856654 | 0.345624 | 0.452401 |
| rule46 | 0.739284 | 0.757098 | 0.399883 | 0.617105 | 0.079279 |
| rule47 | 0.868321 | 0.29095 | 0.401631 | 0.130335 | 0.754388 |
| rule48 | 0.692928 | 0.558599 | 0.781789 | 0.310089 | 0.667824 |
| rule49 | 0.195698 | 0.357847 | 0.281991 | 0.790912 | 0.358728 |
| rule50 | 0.145607 | 0.209672 | 0.963761 | 0.503654 | 0.247264 |
| rule51 | 0.553083 | 0.765271 | 1.383853 | 0.295989 | 0.570781 |
| rule52 | 0.191623 | 0.265268 | 0.300779 | 0.782875 | 0.671055 |
| rule53 | 0.727228 | 0.177754 | 0.545358 | 0.720203 | 0.453408 |
| rule54 | 0.722965 | 0.740491 | 0.41757 | 0.85167 | 0.69817 |
| rule55 | 0.339832 | 0.422281 | 0.590118 | 0.4486 | 0.430758 |
| rule56 | 0.614026 | 0.375941 | 0.965343 | 0.774231 | 0.21888 |
| rule57 | 1.030698 | 0.256162 | 0.22633 | 0.895898 | 0.346057 |
| rule58 | 0.786015 | 0.584065 | 0.105913 | 0.610659 | 0.235599 |
| rule59 | -0.05847 | 0.464951 | 0.63196 | 0.675635 | 0.906144 |
| rule60 | 0.627862 | 0.399215 | 0.648889 | 0.351635 | 0.540221 |
| rule61 | 0.546492 | 0.343717 | 0.588447 | 0.335539 | 0.184862 |
| rule62 | 0.140986 | 0.815063 | 1.001376 | 0.422628 | 0.507533 |
| rule63 | 0.439443 | 0.561035 | 1.115826 | 1.422124 | 0.715875 |
| rule64 | 0.791725 | 0.274637 | 0.129505 | 0.751237 | 0.074114 |
| rule65 | 0.653247 | 0.727141 | 0.512626 | -0.06352 | 0.645469 |
| rule66 | 0.459925 | 0.489808 | 0.49611 | 0.530405 | 0.476276 |
| rule67 | 0.550534 | 1.149042 | 1.013977 | 1.418157 | 0.779472 |
| rule68 | 0.847488 | 0.605599 | 0.430833 | 0.091578 | 0.850205 |
| rule69 | 0.330978 | 0.230283 | 0.062311 | 0.777564 | 0.621303 |
| rule70 | 0.451162 | 0.198432 | 0.539647 | 0.541165 | -0.12088 |
| rule71 | 0.593344 | 0.730746 | 0.348851 | 0.546106 | 0.408322 |
| rule72 | 0.84485 | 0.172377 | 0.147415 | 0.789611 | 0.57423 |
| rule73 | 0.877402 | 0.503185 | 0.265089 | 0.336141 | 0.564005 |
| rule74 | 0.627594 | 0.060641 | 0.631104 | 0.569015 | 0.159324 |
| rule75 | 0.318285 | 0.41702 | 0.188661 | 0.818802 | 0.045192 |
| rule76 | 0.393858 | 0.450039 | 0.636952 | 0.385031 | 0.676705 |
| rule77 | 0.477849 | 0.819584 | 0.491417 | 0.782125 | 0.618623 |
| rule78 | 0.659454 | 0.292013 | 0.520912 | 0.989731 | 0.561013 |
| rule79 | 0.051056 | 0.698918 | 0.251113 | 0.862162 | 1.049352 |
| rule80 | 0.265772 | 0.817804 | 1.126143 | 1.510767 | 0.39945 |
| rule81 | 0.484369 | 0.589235 | -0.01979 | 0.421923 | 0.481006 |
| rule82 | 0.318743 | 0.533833 | 0.452581 | 0.382472 | 0.238906 |
| rule83 | 0.209871 | 0.214848 | 0.357171 | 0.853949 | 0.968735 |
| rule84 | 0.049784 | 0.643548 | 0.752984 | 0.079718 | 0.843925 |
| rule85 | 0.804818 | 1.018202 | 1.116343 | 0.585655 | 0.961342 |
| rule86 | 0.135254 | 0.722275 | 0.750937 | 0.406545 | 0.464431 |
| rule87 | 0.208558 | 0.425764 | 0.925276 | 0.820433 | 0.11912 |
| rule88 | 0.516788 | 0.94207 | 1.04098 | 1.199384 | 0.539884 |
| rule89 | 0.542736 | 0.537528 | 1.180152 | 0.828374 | 0.932607 |
| rule90 | 0.671314 | 0.57764 | 1.005934 | 0.859143 | 0.907157 |
| rule91 | 0.108745 | 0.726079 | 1.022413 | 0.155332 | 0.545775 |
| rule92 | 0.734081 | 0.330248 | 0.523392 | 0.578456 | 0.479727 |
| rule93 | 0.627273 | 0.066513 | 0.764483 | 0.018676 | 0.598536 |
| rule94 | 0.816682 | 0.557913 | 0.926241 | 1.04599 | 0.491827 |
| rule95 | 0.309113 | 0.325803 | 0.362186 | 0.419725 | 0.319224 |
| rule96 | 0.465846 | 0.23077 | 0.392706 | 0.30574 | 0.604993 |
| rule97 | 0.019184 | 0.329684 | 0.225219 | 0.051398 | 0.574166 |
| rule98 | 0.70926 | 0.745275 | 0.120237 | 0.576066 | 0.446271 |
| rule99 | 0.02769 | 0.729773 | 0.3296 | 1.055155 | 0.250526 |
| rule100 | -0.00592 | 0.722066 | 0.533514 | 0.054897 | 0.180086 |
| rule101 | 0.61299 | 0.276181 | 0.432151 | 0.964365 | 0.61087 |
| rule102 | 0.393978 | 0.15914 | 0.100855 | -0.07083 | 0.844787 |
| rule103 | 0.150174 | 0.603862 | 0.606104 | 0.730596 | 1.045714 |
| rule104 | 0.917293 | 0.811642 | 0.206404 | 0.841854 | -0.04239 |
| rule105 | 0.23823 | 0.081645 | 0.466867 | 0.702217 | 0.762494 |
| rule106 | 0.896152 | 0.897471 | 0.388808 | 0.884312 | 1.044085 |
| rule107 | 0.697167 | 0.285053 | 0.406283 | 0.58152 | 0.683676 |
| rule108 | 0.213734 | 0.802264 | 0.43455 | 0.355325 | 0.477339 |
| rule109 | 0.483282 | 0.211509 | 0.379811 | 0.575951 | 0.948939 |
| rule110 | 0.561145 | 0.812944 | 0.507936 | 0.077595 | 0.975243 |
| rule111 | 0.028421 | 0.800632 | 0.301258 | 0.795896 | 0.522683 |
| rule112 | 0.292436 | 0.504283 | 0.143487 | 0.931559 | 1.018348 |
| rule113 | 0.97429 | 0.840139 | 0.151718 | 0.811623 | 0.289693 |
| rule114 | 0.104329 | 0.944557 | 0.2063 | 0.315505 | 0.546477 |
| rule115 | 0.63091 | 0.495399 | 0.910687 | 0.847731 | 0.667007 |
| rule116 | 0.222907 | 0.947613 | 0.051837 | 0.513896 | 0.40646 |
| rule117 | 1.003579 | 0.988965 | 0.207782 | 1.046368 | 0.851803 |
| rule118 | 0.219336 | 0.005138 | 0.058818 | 0.611554 | 0.354736 |
| rule119 | 0.34387 | 0.575318 | 0.434394 | 0.847056 | 0.397105 |
| rule120 | 0.407224 | 0.803403 | 0.096226 | 0.413767 | 0.54815 |
| rule121 | 0.70775 | 0.983243 | 0.834835 | 0.305208 | 0.558642 |
| rule122 | 0.875175 | 0.373821 | 0.654578 | 0.833404 | 0.500715 |
| rule123 | 0.992998 | 0.771465 | 0.439894 | 0.140971 | 0.07685 |
| rule124 | 0.142845 | 0.148552 | 0.141418 | 0.603993 | 0.882034 |
| rule125 | 0.922651 | 0.80176 | 0.827601 | 0.981265 | 0.443057 |
| rule126 | 0.099436 | 0.43608 | 1.019762 | 0.992915 | 0.280691 |
| rule127 | 0.917546 | 0.184614 | 0.406586 | 0.471007 | 0.455267 |
| rule128 | 0.757699 | 0.776775 | 0.515081 | 0.578087 | 0.234517 |
| rule129 | 0.047954 | 0.60107 | 0.564271 | 0.492066 | 0.335002 |
| rule130 | 0.639817 | 0.769837 | 0.044666 | 0.962139 | 0.413103 |
| rule131 | 0.665849 | 0.510502 | 0.586085 | 0.728187 | 0.261712 |
| rule132 | 0.518435 | 0.771178 | 0.325487 | 0.262511 | 0.407755 |
| rule133 | 0.199087 | 0.243063 | 0.296454 | 0.060987 | 0.572213 |
| rule134 | 0.253362 | 0.684186 | 0.048293 | 0.519434 | 0.030942 |
| rule135 | 0.722377 | 0.391324 | 0.871248 | 0.169676 | 0.406744 |
| rule136 | 0.740587 | 0.520255 | 0.139598 | 0.855376 | 0.389171 |
| rule137 | 0.843238 | 0.988904 | 0.256578 | 0.277925 | 0.866742 |
| rule138 | 0.460817 | 0.951896 | 0.573259 | 0.628497 | 0.023905 |
| rule139 | 0.063265 | 0.202415 | 0.512907 | 0.610438 | 0.992849 |
| rule140 | 0.129077 | 0.350881 | 0.62042 | 0.68109 | 0.82064 |
| rule141 | 0.424064 | 0.390391 | 0.357499 | 0.491212 | 0.704056 |
| rule142 | 0.003016 | 0.29208 | 0.040589 | 0.159815 | 0.44147 |
| rule143 | 0.37117 | 0.9473 | 0.557161 | 0.716039 | 0.900072 |
| rule144 | 0.635305 | 0.778237 | 0.849675 | 0.687785 | 0.206005 |
| rule145 | 0.657602 | 0.336005 | 0.568323 | 0.478858 | 0.099595 |
| rule146 | 0.865575 | 0.964117 | 0.301855 | 0.804728 | 0.647579 |
| rule147 | 0.877329 | 0.444876 | 0.577131 | 0.833787 | 0.395462 |
| rule148 | 0.863434 | 0.527854 | 0.290147 | 0.328022 | 1.078513 |
| rule149 | 0.060178 | 0.728686 | 0.433266 | 0.635906 | 0.598342 |
| rule150 | 0.432021 | 0.854879 | 0.70218 | 0.469783 | 0.97849 |
| rule151 | 0.775644 | 0.898081 | 0.280301 | 0.751201 | 0.497864 |
| rule152 | 0.683358 | 0.45353 | 0.749698 | 0.580363 | 0.737391 |
| rule153 | 0.548196 | 0.557985 | 0.808054 | 0.986616 | 0.564081 |
| rule154 | 0.546934 | 0.588664 | 0.944723 | 0.067819 | 0.145811 |
| rule155 | 0.370292 | 0.381728 | 0.709366 | 0.8076 | 0.859828 |
| rule156 | 0.058802 | 0.559329 | 0.469946 | 0.492679 | -0.05949 |
| rule157 | 0.797422 | 0.149581 | 0.219101 | 0.889443 | 0.265188 |
| rule158 | 0.517654 | 0.350691 | 0.757572 | 0.065115 | 0.372651 |
| rule159 | 0.497563 | 0.411005 | 0.802262 | 0.856048 | 0.257874 |
| rule160 | 0.709665 | 0.658796 | 0.213929 | 0.50281 | 0.13291 |
| rule161 | 0.017588 | 0.25296 | 0.271126 | 0.39967 | -0.08666 |
| rule162 | 0.738399 | 0.724209 | 0.379092 | 0.587959 | 0.137481 |
| rule163 | 0.411407 | 0.504483 | 0.984355 | 0.054932 | 0.098076 |
| rule164 | 0.995091 | 0.686217 | 0.310613 | 0.887946 | 0.992194 |
| rule165 | 0.267061 | 0.952425 | 0.716349 | 0.516644 | 0.474661 |
| rule166 | 0.801805 | 0.42817 | 0.258226 | 0.767611 | 0.702713 |
| rule167 | 0.729098 | 0.020312 | 0.146317 | 0.789705 | 0.477274 |
| rule168 | 0.15322 | 0.89065 | 0.567911 | 0.329944 | 0.388958 |
| rule169 | 0.197699 | 0.539256 | 0.071556 | 0.09564 | 0.185483 |
| rule170 | 0.735088 | 0.491843 | 0.660396 | 0.488934 | 0.02076 |
| rule171 | 0.488136 | 0.453889 | 0.085279 | 0.696769 | 0.468574 |
| rule172 | 0.599427 | 0.719189 | 0.789491 | 0.040867 | 0.191347 |
| rule173 | 0.832365 | 0.803908 | 0.551915 | 0.934337 | 0.81993 |
| rule174 | 0.575267 | 0.131369 | 0.013446 | 0.354325 | 0.817822 |
| rule175 | 0.3196 | 0.070525 | 0.027346 | 0.535203 | 0.231197 |
| rule176 | 0.354807 | 0.054105 | 0.074034 | 0.304565 | 0.72267 |
| rule177 | 0.921 | 0.951704 | 0.055893 | 0.437245 | 0.684778 |
| rule178 | 0.435546 | 0.266007 | 0.007266 | 0.122337 | 0.838051 |
| rule179 | 0.359876 | 0.084389 | 0.652676 | 0.111837 | 0.083516 |
| rule180 | 0.574667 | 0.968465 | 0.988367 | 0.359502 | 0.688754 |
| rule181 | 0.369674 | 0.934281 | 0.892211 | 0.640171 | 0.918309 |
| rule182 | 0.912216 | 0.863562 | 0.020988 | 0.092147 | 0.160088 |
| rule183 | 0.644438 | 0.316598 | 0.505288 | 0.569678 | 0.566854 |
| rule184 | 0.867501 | 0.142921 | 0.818562 | 0.033571 | 0.812078 |
| rule185 | 0.000642 | 0.275571 | 0.265659 | 0.982813 | 0.901653 |
| rule186 | 0.187034 | 0.087334 | 0.157935 | 0.221658 | 0.267184 |
| rule187 | 0.61672 | 0.103255 | 0.289266 | 0.371908 | 0.507444 |
| rule188 | 0.200001 | 0.072509 | 0.902741 | 0.689803 | 0.828619 |
| rule189 | 0.954574 | 0.718731 | 0.870528 | 0.37079 | 0.396648 |
| rule190 | 0.42777 | 0.24291 | 0.768513 | 0.449121 | 0.206469 |
| rule191 | 0.990945 | 0.942687 | 0.963984 | 0.91202 | 0.287073 |
