# Supplementary material for: On the combination of adaptive neuro-fuzzy inference system and deep residual network for improving detection rates on intrusion detection
Source: PLoS One. 2022 Dec 12;17(12):e0278819. doi: 10.1371/journal.pone.0278819 (PMC9744302; doi:10.1371/journal.pone.0278819)
Supplement: S2 Table — (DOCX) [file pone.0278819.s003.docx]

**S1 Table.** **Correlation Degree of 41 Attributes towards Target**

| Attribute | Correlation Degree |
| --- | --- |
| dst_host_srv_count | -0.62425 |
| logged_in | -0.57604 |
| hard | -0.557252 |
| dst_host_same_srv_rate | -0.525837 |
| same_srv_rate | -0.510869 |
| flag | -0.501333 |
| srv_count | -0.038856 |
| num_access_files | -0.030234 |
| su_attempted | -0.019483 |
| num_file_creations | -0.014385 |
| num_root | -0.01002 |
| num_compromised | -0.009003 |
| is_host_login | -0.002334 |
| land | 0.002944 |
| urgent | 0.003941 |
| num_shells | 0.005208 |
| root_shell | 0.007757 |
| dst_bytes | 0.008651 |
| srv_diff_host_rate | 0.010454 |
| src_bytes | 0.012981 |
| num_failed_logins | 0.02312 |
| is_guest_login | 0.0306 |
| wrong_fragment | 0.054795 |
| hot | 0.065085 |
| duration | 0.129443 |
| protocol_type | 0.168167 |
| dst_host_count | 0.196101 |
| service | 0.233783 |
| dst_host_srv_diff_host_rate | 0.239645 |
| diff_srv_rate | 0.282313 |
| dst_host_rerror_rate | 0.293804 |
| rerror_rate | 0.308667 |
| srv_rerror_rate | 0.309907 |
| dst_host_srv_rerror_rate | 0.310226 |
| dst_host_same_src_port_rate | 0.319097 |
| srv_serror_rate | 0.378666 |
| serror_rate | 0.381644 |
| dst_host_serror_rate | 0.382115 |
| dst_host_srv_serror_rate | 0.384793 |
| count | 0.388355 |
| dst_host_diff_srv_rate | 0.391327 |
